# Advances in Uncertain Information Fusion

**DOI:** 10.3390/e26110945

**Published:** 2024-11-05

**Authors:** Lianmeng Jiao

**Affiliations:** School of Automation, Northwestern Polytechnical University, Xi’an 710072, China; jiaolianmeng@nwpu.edu.cn

## 1. Introduction

Information fusion is the combination of information from multiple sources, which aims to draw more comprehensive, specific, and accurate inferences about the world than are achievable from the individual sources in isolation [1,2]. This technique is widely applied in many areas, such as target tracking and recognition in battlefield surveillance [3,4], sensor fusion in robotics [5], image fusion in computer vision [6], expert opinion fusion in risk analysis [7], and so on. Since the sensory data are inherently noisy, and human knowledge is inevitably imprecise, ambiguous, or irrelevant, the right handling of such uncertain information is always at the core of any fusion system [8]. This gives rise to a series of both theoretical and practical challenges with focuses on two aspects: how the uncertainty is expressed or quantified, and how uncertain pieces of information can be aggregated. Possible approximate reasoning theories for managing uncertain information include, but are not limited to, Bayesian probability theory, fuzzy sets, random sets, rough sets, possibility theory, and Dempster–Shafer evidence theory [9]. Consequently, this Special Issue focuses on the latest advances in uncertain information fusion by modeling and reasoning uncertain information with various approximate reasoning theories.

This Special Issue features six articles that explore various theories for uncertain information fusion from an information-theoretical perspective. Specifically, two articles focus on Dempster–Shafer evidence theory [10,11], one on fuzzy set theory [12], one on possibility theory [13], and two on classical Bayesian theory [14,15]. Detailed descriptions of these articles are provided below.

## 2. An Overview of Published Articles

The first article [10] of this Special Issue focuses on measuring the uncertainty in the negation evidence for the multi-source information fusion problem. The negation evidence is developed based on the Dempster–Shafer evidence theory, which is widely used in modeling and reasoning uncertain information in many real-world applications. However, how to address the uncertainty in the negation information modeled as the negation of the basic probability assignment (BPA) is still an open issue. Inspired by the uncertainty measures in Dempster–Shafer evidence theory, a method of measuring the uncertainty in the negation evidence is developed in this article. The authors first propose a new measure to quantify the uncertainty of the negation evidence by extending the classical belief entropy, and then develop an improved multi-source information fusion method considering uncertainty quantification in the negation evidence with the new measure. Finally, experiments based on a numerical example and a fault diagnosis problem are conducted to verify the rationality and effectiveness of the proposed method in measuring and fusing uncertain information.

Similar to the above work, the second article [11] of this Special Issue also investigates the uncertain information fusion problem based on the Dempster–Shafer evidence theory. This article focuses on measuring the correlation between belief functions, which can provide a more comprehensive reference for uncertain information processing. The authors first propose a new correlation measure, called the belief correlation measure, based on the belief entropy and the relative entropy. This measure takes into account the influence of information uncertainty on their relevance, and possesses several important mathematical properties, such as probabilistic consistency, non-negativity, non-degeneracy, boundedness, orthogonality, and symmetry. Furthermore, an information fusion method is developed based on the new belief correlation measure, which introduces both the objective weight and the subjective weight to assess the credibility and usability of belief functions, thus providing a more comprehensive measurement for each piece of evidence. At last, two target classification tasks are considered to demonstrate the effectiveness of the proposed method.

Nieto-Morote et al. [12] focus on modeling the uncertain information with fuzzy set theory. Specifically, this article investigates the membership function assignment problem based on inherent features of linguistic terms to determine their semantics when they are used for preference modeling. In their work, the authors uphold that the required mathematical function assignment to a linguistic term should be based on linguistic semantics and pragmatic principles. Consequently, two different membership function assignment rules are developed: the fuzzy relational calculus, and the horizon shifting model derived from the alternative set theory, which are used to handle weakening and reinforcement hedges, respectively. The proposed elicitation method provides for the term set semantics, non-uniform distribution of non-symmetrical triangular fuzzy numbers, depending on the number of terms used and the character of the hedges involved.

Chen et al. [13] study the autonomous search for targets in the framework of possibility theory, which provides a means for quantitative modeling and reasoning in the presence of epistemic uncertainty. Autonomous search is an ongoing cycle of sensing, statistical estimation, and motion control with the objective to find and localize targets in a designated search area. The traditional theoretical framework for autonomous search usually combines sequential Bayesian estimation with information theoretic motion control. This work formulates autonomous search problem in the framework of possibility theory, where the detection probability is partially known, and expressed as an interval value. The authors develop an elegant Bayes-like solution to sequential estimation, with the reward function for motion control defined to take into account the epistemic uncertainty. The numerical results demonstrate that the proposed possibilistic formulation of search can deal effectively with epistemic uncertainty in the form of interval-valued probability of detection.

Liu et al. [14] investigate the multi-modal fusion for emotion recognition in the classical Bayesian framework. In this work, a parallel, multi-modal, factorized, and bilinear pooling method is proposed based on a semi-tensor product (STP) for information fusion in emotion recognition. First, the STP is used to factorize a high-dimensional weight matrix into two low-rank factor matrices without dimension matching constraints. Then, the multi-modal features are projected to the low-dimensional matrices and multiplied based on the STP to capture the rich interactions between the features. Finally, an STP-pooling method is used to reduce the dimensionality to obtain the final features. This method can achieve the information fusion between modalities of different scales and dimensions, and avoids data redundancy due to dimension matching. The experimental results on benchmark emotion-recognition tasks validate that the proposed method improves the performance and reduces both the training time and the number of parameters.

The final article [15] of this Special Issue explores the feasibility of combining a specialized segmentation model with a zero-shot segmentator like the Segment-Anything Model (SAM) in the classical Bayesian framework. SAM is a promptable segmentation system that offers zero-shot generalization to unfamiliar objects and images, eliminating the need for additional training. In this work, the authors propose to improve the segmentation performance by providing SAM with checkpoints extracted from the masks produced by mainstream segmentators, and then merging the segmentation masks provided by these two networks. Exhaustive tests are conducted on seven heterogeneous public datasets, and the experimental results show that a combination of segmentators at the logit level can lead to segmentation improvements over the original masks obtained by mainstream segmentators, even beating the state-of-the-art segmentators on some datasets. The results of this work provide valuable insights into the potential of incorporating the SAM segmentator with existing segmentation techniques.

## 3. Conclusions

This Special Issue presents six contributions covering various approximate reasoning theories for modeling and reasoning uncertain information with applications in a wide range of fields including fault diagnosis, target classification, emotion recognition and image segmentation. Current trends indicate that representation and qualification of various uncertainty will remain dominant research directions for next-generation information fusion systems. It is also evident that deep learning or foundation models (such as SAM) are becoming increasingly common for designing information fusion methods, as multi-modal data (such as image, speech, and text) are usually collected in many real-world applications. Therefore, integrating the classical approximate reasoning theories with the advanced deep learning models can potentially become more important in future uncertain information fusion systems.
